# Comparison of the clinical outcomes of skin bridge loop ileostomy and traditional loop ileostomy in patients with low rectal cancer

**DOI:** 10.1038/s41598-021-88674-x

**Published:** 2021-04-27

**Authors:** Hui Ye, Shujuan Huang, Jie Yu, Qichang Zhou, Changlei Xi, Longlei Cao, Peiyun Wang, Jie Shen, Zhilin Gong

**Affiliations:** 1 Department of Colorectal Anal Surgery, Jingzhou Central Hospital, The Second Clinical Medical College, Yangtze University, No. 60 Jingzhong Road, Jingzhou, 434020 Hubei Province China; 2 Department of Respiratory and Critical Care Medicine, Jingzhou Central Hospital, The Second Clinical Medical College, Yangtze University, Jingzhou, 434020 Hubei Province China

**Keywords:** Gastrointestinal diseases, Gastrointestinal cancer

## Abstract

To compare the clinical results of patients with low rectal cancer who underwent skin bridge loop ileostomy and traditional loop ileostomy, and provide clinical evidence for choosing a better ostomy method. We retrospectively collected data of 118 patients with rectal cancer who underwent low anterior resection and loop ileostomy. To investigate the patients characteristics, postoperative stoma-related complications and the frequency of exchanged ostomy bags. The differences of these indicators between the two groups of patients who underwent skin bridge loop ileostomy and traditional loop ileostomy were compared. The Visual Analog Scale (VAS) score of the skin bridge loop ileostomy group was lower than that of the traditional ileostomy loop group (*P* < 0.05). The skin bridge group had a lower Discoloration, Erosion, Tissue overgrowth (DET) score and incidence of mucocutaneous separation than the traditional group at the 1st and 2nd weeks after operation (*P* < 0.05). The average number of weekly exchanged ostomy bags was significantly less in the skin bridge group than in the traditional group within 4 weeks after surgery (*P* < 0.05). Our experience demonstrates that the skin bridge loop ileostomy may significantly reduce early postoperative stoma-related complications, the frequency of exchanged ostomy bags and patients’ medical costs after discharge.

## Introduction

Colorectal cancer is a common tumor of the digestive system. This is 3rd in men and 2nd in women in terms of tumor incidence according to reports^1,2^. With the clinical application of neoadjuvant chemoradiotherapy and the development of minimally invasive surgical techniques, the possibility of anus preservation in patients with low rectal cancer is increasing. But anastomotic leakage is one of the most serious complications in this operation, and it is more common in surgery of low anterior resection or intersphincteric resection for low rectal cancer^3,4^. Stoma is considered to be an effective method in order to prevent this complication^5,6^. But it may also cause stoma-related complications, such as peristomal dermatitis and retraction, some of which are common in clinic and significantly affect postoperative quality of life of the patients^7,8^.

Therefore, how to reduce the complications associated with the stoma has become a problem worthy of surgeons' attention. Stoma rods are used traditionally to prevent retraction of loop stoma into the abdominal cavity. But recent systematic reviews and meta-analyses have already showed that stoma rods do not reduce the incidence of stoma retraction and instead lead to increased rates of stoma-related complications^9,10^.

Skin bridge loop ileostomy have been reported by some experts, which used a skin bridge instead of a support rod^11^. In recent years, we have made this stoma in low or ultra-low anterior rectal resection and achieved satisfactory clinical outcomes compared with the traditional loop ileostomy.

## Results

### Patient characteristics

A total of 95 patients were included in this study, of these, 42 underwent skin bridge loop ileostomy and 53 underwent traditional loop ileostomy, and 23 were excluded from the study due to incomplete data. There were no significant differences in the data of patients characteristics including sex, age, The American Society of Anesthesiologists classification of physical status(ASA-PS), tumor stage, Body mass index (BMI), operation type, history of diabetes mellitus, operation time and postoperative length of hospital stay between two groups (*P* > 0.05) (Table 1).

**Table 1:** Patient characteristics.

characteristics	Skin bridge (*N* = 42)	Conventional (*N* = 53)	χ^2^	*t*	*P*
Sex, male/female	23/19	31/22	0.13		0.72
Age, years	59.90 ± 12.07	59.53 ± 12.19		0.15	0.88
Stage			0.94		0.63
I	7	6			
II	20	30			
III	15	17			
ASA-PS			0.81		0.67
1	16	16			
2	20	30			
3	6	7			
BMI, kg/m^2^	22.70 ± 2.28	22.53 ± 2.47		0.34	0.74
Operation type			0.04		0.84
Low anterior resection	35	45			
Intersphincteric resection	7	8			
Diabetes mellitus	5	8	0.20		0.65
Operation time, min	167.81 ± 29.84	169.72 ± 28.38		0.32	0.75
Postoperative length of hospital stay, days	9.88 ± 1.53	10.26 ± 1.83		1.09	0.28

### Complications and number of weekly exchanged ostomy bags of creation of skin bridge and conventional loop ileostomy

The VAS score of the skin bridge loop ileostomy group was lower than that of the traditional ileostomy loop group at a week after operation [(0.76 ± 0.66) vs. (2.49 ± 1.42), *P* < 0.05]. The skin bridge loop ileostomy group had a lower DET score [(0.86 ± 1.07) vs. (3.21 ± 2.27), *P* < 0.05; (1.79 ± 1.49) vs. (6.40 ± 3.52), *P* < 0.05] and incidence of mucocutaneous separation [4.76% (2/42) vs. 18.87% (10/53), *P* < 0.05; 11.90% (5/42) vs. 60.38% (32/53), *P* < 0.05] than the traditional group at the 1st and 2nd weeks after operation, and there were no differences at the 4th week after operation and before stoma closure (*P* > 0.05). The complication of parastomal hernias between two groups was no significant difference (*P* > 0.05). The average number of weekly exchanged ostomy bags was significantly less in the skin bridge loop ileostomy group than in the traditional loop ileostomy group within 4 weeks after surgery [(1.38 ± 0.49) vs. (2.36 ± 0.92), *P* < 0.05]. There were no cases of stoma prolapse, retraction, stenosis and necrosis after operation in both groups (Table 2).

**Table 2:** Stoma-related complications and number of weekly exchanged ostomy bags of creation of skin bridge versus conventional ileostomy.

	Skin bridge (*N* = 42)	Conventional (*N* = 53)	^χ2^	*t*	*P*
VAS score	0.76 ± 0.66	2.49 ± 1.42		7.28	0.00
**DET score**					
I week after operation	0.86 ± 1.07	3.21 ± 2.27		6.12	0.00
2 week after operation	1.79 ± 1.49	6.40 ± 3.52		7.95	0.00
4 week after operation	0.67 ± 1.12	0.81 ± 1.23		0.59	0.55
Before stoma closure	0.60 ± 1.04	0.66 ± 1.11		0.29	0.77
**Mucocutaneous separation**					
I week after operation	2	10	4.23		0.04
2 week after operation	5	32	23.15		0.00
4 week after operation	1	5	–		0.22
Before stoma closure	0	0	–		–
Parastomal hernias	8	13	0.41		0.52
Stoma prolapse	0	0	–		–
Stoma retraction	0	0	–		–
Stoma stenosis	0	0	–		–
Stoma necrosis	0	0	–		–
Number of exchanged ostomy bags/week	1.38 ± 0.49	2.36 ± 0.92		6.20	0.00

## Discussion

Low rectal cancer generally refers to a rectal malignant tumor located below the peritoneal reflection and within 5 cm of the dentate line. Anastomotic leakage is one of the most serious complications of low anterior rectal resection. It has a higher incidence in elderly patients or who with preoperative neoadjuvant chemoradiotherapy, and the rate is 5.8–18.6% according to the clinical reports^12,13^. Stoma can decrease the occurrence of anastomotic leakage with reducing the local pressure and pollution, and promoting healing^14,15^.

However, the stoma changes the normal passage and excretion of intestinal contents and may cause some complications, which will affect patient's postoperative recovery and quality of life^16,17^. In order to reduce the incidence of stoma-related complications, surgeons are constantly improving the way in which the stoma is made. The colostomy operation is more complicated and the time is more longer than the ileostomy, especially during the operation of stoma closure, and it has been reported that there are more postoperative complications^18–20^, so the ileostomy is gradually increasing in clinic at present.

Traditional loop ileostomy need to use a hard plastic rod to support the ileal wall to prevent stoma retraction^21,22^. The rod will be removed about 2 weeks after the operation. During this period, the compressed skin under the rod is prone to inflammatory reactions such as local erosion and ulcers, leading to pain and other discomfort, which affects out-of-bed activity in patients. At the same time, the support rod will affect the stoma care procedure. The dermatitis around the stoma makes the ostomy bag inadequately adhered, which causes fecal leakage to further aggravate the dermatitis and the need for frequent change of the bag, especially for beginner. What's more, the rod causes the skin below and near it to be misaligned and leave gap, so many patients have mucocutaneous separation. It can exacerbate peristomal dermatitis and even cause wound infection. Some scholars^23,24^ have used pedicle flap to make skin bridge instead of support rods in order to reduce stoma-related complications, but its advantages and disadvantages still need to be further evaluated.

We retrospectively analyzed and summarized the skin bridge loop ileostomy operated in our hospital, and compared with the traditional loop ileostomy. The former not only doesn’t require a support rod locally, but also ensures the safety of operation and reduces stoma-related complications. This method doesn’t increase the operation time, and is easy to learn and popularize. In addition, patients do not need to return to the hospital for rod removal at 2 weeks after surgery, which can reduce the cost of medical treatment for patients and decrease the workload of medical care for doctors and nurses. The results of this study showed that compared with the traditional loop ileostomy group, in the skin bridge loop ileostomy group, there has a lower VAS and DET score at a week after surgery, also a lower DET score at two weeks(*P* < 0.05) (Table 2), the incidence of stoma mucocutaneous separation decreased significantly at two weeks (*P* < 0.05) (Table 2), and the number of exchanged ostomy bag decreased within 4 weeks(*P* < 0.05). There are three reasons. Firstly, in the skin bridge loop ileostomy group, the stoma made on the pedicle flap that is at the same level as the surrounding skin and will not stress other tissues. Secondly, there is no need to use a support rod, which doesn’t affect the patient's early activity and the stoma care procedure, and the sticking of the ostomy bag will be more tight. Thirdly, A flap can form a good fit with the intestine, leaving no gap. The support rod will be removed in the traditional loop iliostomy group at 2 weeks after the operation and the pain, dermatitis and mucocutaneous separation will be gradually disappeared. The DET score and the incidence of mucocutaneous separation had no significant differences at 4 weeks after operation and before stoma closure(*P* > 0.05). It indicates that the skin bridge loop ileostomy mainly reduces early postoperative complications and has no effect on the long-term outcomes. Further more, the results of this study showed that there were no cases of stoma prolapse, retraction, stenosis and necrosis in the two groups after surgery, and the complication of parastomal hernias wasn’t significant different. So in terms of skin bridge loop ileostomy, it is as safe and reliable as traditional loop ileostomy.

Of course, sometimes stoma stenosis may occur when the intestine is pulled out of the abdominal wall due to the differences in individual characteristics of patients, such as the degree of obesity, the thickness of intestine and mesentery, and the elasticity of skin. Our approach to this problem is to make a longer (approximately 3.0 cm) and narrower (approximately 1.0 cm) flap and crescent-shaped skin resection on the upper and lower incision of stoma when patient have a fatter body, thicker intestine and mesentery, and less elastic skin. Moreover, because the main direction of the distribution of vessels and nerves in the anterior abdominal wall is from the outside to the inside, we designed the skin flap as transverse rectangular bridge with it’s pedicle on the outside to reduce vascular and nerval damage and avoid stoma retraction caused by flap necrosis.

In summary, we believe that the skin bridge loop ileostomy is better than the traditional loop ileostomy, which may reduce the early postoperative complications related to the stoma, avoid to remove the stoma support rod at 2 weeks after operation, decrease the number of exchanged ostomy bag and save the patient's expenses. We think that it has certain application value in clinic. Further studies are required to confirm the true benefits of the skin bridge loop ileostomy.

## Method

### Patients

We retrospectively collected data of 118 patients with rectal cancer who underwent low anterior resection and loop ileostomy between January 2015 to December 2019 at the Department of Colorectal Anal Surgery of the Second Clinical Medical College, Yangtze University, China. Inclusion criteria were patients with stage I–III tumors according to American Joint Committee on Cancer (AJCC) 8th edition, the location of tumor within 5 cm from the dentate line and they with stage ≥ T3 were received preoperative neoadjuvant therapy. Exclusion criteria were open surgery, emergency surgery, ASA-PS > 3, inflammatory bowel disease and neuropsychiatric disorder. Abdominal stoma positioning was performed by enterostomal therapist before surgery. Our study was approved by the institutional review board for studies in humans.

Clinicopathological parameters including sex, age, ASA-PS, tumor stage, BMI, operation type, history of diabetes mellitus, operation time, postoperative length of hospital stay, average number of weekly exchanged ostomy bags and postoperative complications related to stoma were evaluated by reviewing medical and pathology reports. The complications included pain, peristomal dermatitis, mucocutaneous separation, prolapse, retraction, stenosis and necrosis. The severity of pain and peristomal dermatitis were evaluated by VAS and DET score.

### Surgical techniques

#### Skin bridge loop ileostomy group

**Figure 1:**
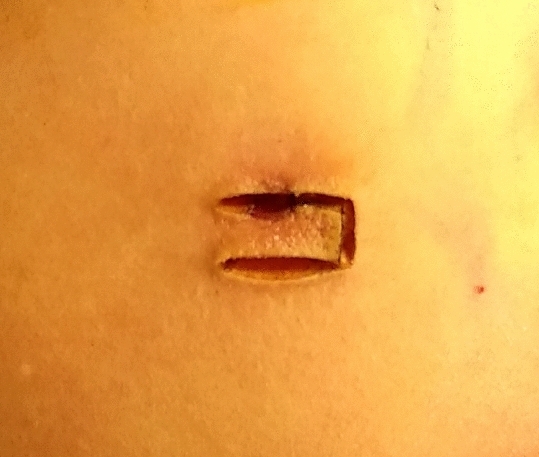
The skin was incised to create a rectangular skin bridge 2.5–3 cm long and 1–1.5 cm wide.

**Figure 2:**
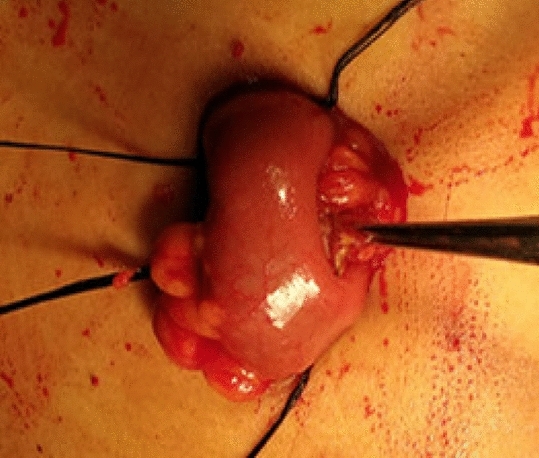
The ileal loop was withdrawn through the opening of the abdominal wall. The intestinal wall and mesentery were intermittently sutured with peritoneum and external oblique aponeurosis. An avascular window was opened in the mesentery and the skin bridge was passed through it. Then the bridge was secured with 2–3 stitches of 2/0 absorbable suture to the distal edge of the opening.

**Figure 3:**
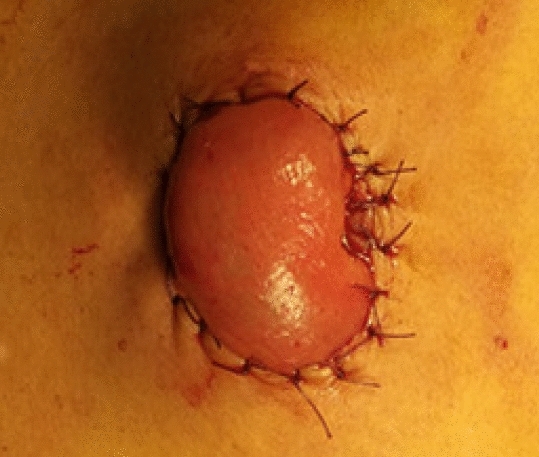
Both loops were fixed to the skin with 3/0 absorbable suture.

The stoma site was marked preoperatively and it was the same as one trocar site in order to reduce a surgical incision. Low anterior rectal resection was completed with conventional laparoscopic surgery. The skin was incised at the pre-marked site of stoma to create a rectangular skin bridge 2.5–3 cm long and 1–1.5 cm wide (Fig. 1). The subcutaneous fat was removed. A longitudinal linear incision was made in the external oblique aponeurosis, obliquus internus abdominis and transversus abdominis were separated, and then the peritoneum was opened. The ileal loop was withdrawn through the opening of the abdominal wall and 2–3 cm above the skin surface. The intestinal wall and mesentery were intermittently sutured with peritoneum and external oblique aponeurosis with absorbable sutures to prevent parastomal hernias. An avascular window was opened in the mesentery which was adjacent to the ileal wall and 20 cm away from ileocecus, and the skin bridge was passed through it. Then the bridge was secured with 2–3 stitches of 2/0 absorbable suture to the distal edge of the opening (Fig. 2). Both loops were fixed to the skin with 3/0 absorbable suture (Fig. 3). The stoma was opened along the longitudinal axis of the intestinal wall and the ostomy bag was stuck.

#### Traditional loop ileostomy group

**Figure 4:**
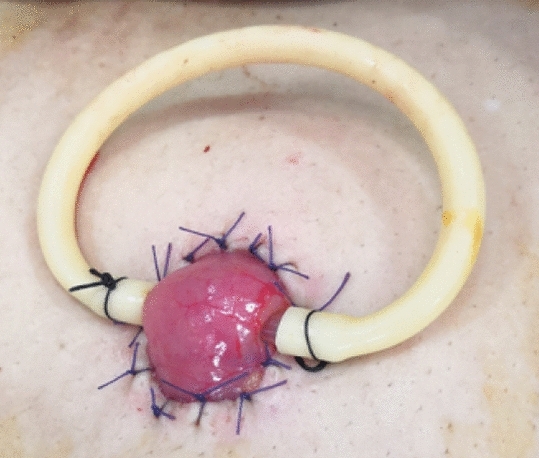
Appearance of the traditional loop ileostomy.

A circular incision with a diameter of 2.5–3.0 cm was made at the planned stoma site, and the skin and subcutaneous fat were removed. After opening the peritoneum, The ileal loop was pulled out from here. A plastic rod was passed through the avascular area of mesentery to fix ileal wall and prevent the stoma retraction (Fig. 4). The rest of the procedure was the same as that of the skin bridge loop ileostomy group. The plastic rod was removed at 2 weeks after the operation. Patients in both two groups were accepted stoma closure operation at 3–6 months after this surgery.

### Patient follow-up

The patient's VAS Score was completed and recorded by the nurse in the inpatient department at a week after operation. If there were no serious complications, the patient follow-up was completed by the doctor and enterostomal therapist until the stoma was closed in the outpatient department. Enterostomal therapist assessed the degree of peristomal dermatitis by using the DET Score at four time points. The 1st time point was a week after the operation in the inpatient department. The 2nd was the 1st outpatient review at 2 weeks after the operation. The 3rd was 4 weeks after the operation. The 4th was just before stoma closure. The mucocutaneous separation and other complications including stoma prolapse, retraction, stenosis, necrosis and parastomal hernias were also recorded at the above time points.

### Statistical analysis

Statistical analyses were performed using SPSS 19.0 software. Statistically significant differences were determined by Student’s t test or Fisher’s exact test as appropriate. Probabilities of < 0.05 were considered significant.

### Ethics approval and consent to participate

The Institutional Review Board (IRB) of Jingzhou Central Hospital reviewed the protocol. All methods were performed in accordance with the relevant guidelines and regulations and the IRB approved the protocol. This is a retrospective study and the IRB waived the need for written informed consent.
